# Does exercising bilaterally require measuring bilaterally? Bilateral versus unilateral tourniquet cuff inflation during measurement of arterial occlusion pressure: considerations for blood flow restriction exercise

**DOI:** 10.3389/fphys.2026.1789582

**Published:** 2026-06-05

**Authors:** Robert Bielitzki, Tom Behrendt, Martin Behrens, Jan Peter Latendorf, Alexander Franz, Luke Hughes, Lutz Schega

**Affiliations:** 1 Department of Human Movement Science and Exercise Physiology, Institute of Human Movement Science, University of Hamburg, Hamburg, Germany; 2 ProPrevent GbR, Magdeburg, Germany; 3 University of Applied Sciences for Sport and Management Potsdam, Potsdam, Germany; 4 Department of Orthopaedics, Rostock University Medical Center, Rostock, Germany; 5 Chair of Health and Physical Activity, Department of Sport Science, Institute III, Otto-von-Guericke University Magdeburg, Magdeburg, Germany; 6 Department of Orthopedics and Trauma Surgery, University Hospital Bonn, Bonn, Germany; 7 Aerospace Medicine and Rehabilitation Laboratory, School of Sport Exercise and Rehabilitation, Northumbria University, Newcastle upon Tyne, United Kingdom

**Keywords:** blood flow restriction, body position, limb occlusion pressure, pneumatic tourniquet cuff, vascular occlusion

## Abstract

**Introduction:**

It is recommended to use a tourniquet cuff pressure between 40-80% of the individuals’ arterial occlusion pressure (AOP) during blood flow restriction (BFR) exercise. The AOP is usually determined in one limb for unilateral BFR exercise or in both limbs individually for bilateral BFR exercise. However, given that the tourniquet cuffs are inflated at both limbs simultaneously during bilateral BFR exercises (e.g., cycling, walking, and squat exercise), it is currently not known if the respective AOP also needs to be determined during bilateral tourniquet cuff inflation. Consequently, the present study aimed to compare the AOP during unilateral versus bilateral tourniquet cuff inflation in the lower extremities.

**Methods:**

In a randomized cross-over trial, the AOP of 25 young healthy participants was determined during unilateral and bilateral tourniquet cuff inflation in supine, seated, and standing position. All measurements were completed in one experimental session with a rest period of 5 min in between. At the beginning and end of each condition, heart rate and blood pressure were recorded.

**Results:**

Regardless of body position, AOP was higher during bilateral compared to unilateral tourniquet cuff inflation (*mean difference* = 3.2 mmHg [*95% confidence interval*: 1.0, 5.4], *p* = 0.006, *d* = 0.12). Furthermore, AOP and heart rate increased with change in body position from supine to seated to standing position (*p* < 0.001, *d* ≥ 0.76).

**Discussion:**

Even though there was a statistically significant difference in AOP between unilateral and bilateral tourniquet cuff inflation irrespective of body position, bilateral cuff inflation during AOP determination appears to have only minor impact on AOP in the lower extremities given the small mean difference and trivial effect size. However, since differences in AOP between unilateral and bilateral tourniquet cuff inflation of ≥ 30 mmHg have been recorded, practitioners must be aware of potentially pronounced differences between unilateral and bilateral cuff inflation during AOP determination in some individuals using the specific BFR device and AOP measurement protocol employed in this study. These results need to be verified for different inflation protocols, devices (i.e., manual versus automatic AOP determination), and populations (e.g., older adults, patients).

## Introduction

Blood flow restriction (BFR) exercise is usually performed with low external loads and requires the application of a pneumatic or non-pneumatic tourniquet cuff proximally at the limb to reduce arterial blood flow and fully occlude venous return in distal tissues (Mattocks et al., 2018). Low external load exercise combined with BFR has been shown to increase the psychophysiological response (i.e. internal load) compared to external load-matched exercise without BFR (Bielitzki et al., 2024). For instance, low external load BFR exercise has been shown to elicit higher acute responses of internal load (e.g., myoelectrical activity (Husmann et al., 2018; Ilett et al., 2019) compared to the same exercise without BFR as well as reduced external load (e.g., exercise volume (Jessee et al., 2019; Pignanelli et al., 2020)) compared to load-matched exercise without BFR to volitional failure. Furthermore, low external load resistance training combined with BFR can induce chronic adaptations similar to conventional high external load resistance training (e.g., muscle mass (Lixandrão et al., 2018)). Existing literature suggests that acute responses and chronic adaptations to BFR exercise are determined by the tourniquet cuff pressure (Bielitzki et al., 2024) and it is recommended to use pressures between 40-80% of the individual’s arterial occlusion pressure (AOP) for safe and effective BFR exercise (Patterson et al., 2019). When determining the AOP, there are several influencing factors that have to be considered (Queiros et al., 2023) such as limb circumference, systolic blood pressure (Loenneke et al., 2015), body position (Hughes et al., 2018), and circadian rhythm (Ingram et al., 2017), while studies have not revealed differences in AOP between the left and right lower limb (Evin et al., 2021; Hughes et al., 2021). Commonly, AOP measurements are individually performed for each leg meaning with unrestricted blood flow to the contralateral limb (unilateral tourniquet cuff inflation), even when the BFR exercise was performed with tourniquet cuffs inflated at both limbs (bilateral tourniquet cuff inflation). However, it remains unclear whether the AOP in one limb is influenced when tourniquet cuffs are inflated at both limbs and if it is thus necessary to determine the AOP during bilateral tourniquet cuff inflation for bilateral BFR exercise. Therefore, this study investigated whether unilateral tourniquet cuff inflation versus bilateral tourniquet cuff inflation during AOP determination would lead to differences in AOP in the lower extremities. Given that the AOP is usually determined during different body positions due to hemodynamic changes, the AOP was determined in supine, seated, and standing position (Sieljacks et al., 2018).

Furthermore, Partsch and Partsch (2005) have determined the calf compression pressure to fully occlude superficial and deep legs veins in supine (20–25 mmHg), seated (50–60 mmHg), and standing position (~70 mmHg) in 9 healthy participants and 5 patients with small saphenous veins. Therefore, it was expected that blood flow in the femoral veins would be blocked at earlier stages during the AOP determination (i.e., gradual increase of the tourniquet cuff pressure until arterial blood flow is fully occluded). The venous occlusion is thought to cause venous blood pooling (Takarada et al., 2000; Moore et al., 2004) and might lead to distension, which has been shown to activate group IV afferent muscle fibers (Haouzi et al., 1999; Husmann et al., 2018). It is established that the neural feedback originating from the afferent nerves of groups III/IV within the muscles amplifies cardiovascular responses, a phenomenon known as mechano-metaboreflex (Angius et al., 2022). Since studies have shown no differences in AOP between legs (*p* = 0.730 (Evin et al., 2021), *p* > 0.05 (Hughes et al., 2021)) it can be speculated that the venous expansion will be similar in both limbs at the same time during bilateral tourniquet cuff inflation. Therefore, cardiovascular responses (i.e., heart rate and blood pressure) are assumed to increase during AOP determination with bilateral tourniquet cuff inflation. Since systolic blood pressure is known as an influencing factor for AOP (Loenneke et al., 2015; Brown et al., 2018), it was hypothesized that bilateral tourniquet cuff inflation during AOP measurement leads to a higher AOP compared with unilateral tourniquet cuff inflation.

## Methods

### Participants

For the primary outcome (i.e., AOP), a sample size calculation with an assumed medium effect size (f = 0.25) for a 2 × 3 (inflation condition [uni-, bilateral] × body position [supine, seated, standing]) repeated measures analysis of variance (rmANOVA) with α = 0.05, 1-β = 0.90, and a correlation among repeated measures = 0.7 (Karanasios et al., 2022) was performed using G*Power (Version 3.1.9.7, Heinrich-Heine University Düsseldorf, Germany) revealing a required sample size of n = 22. Participants with (i) hypertension (> 140/90 mmHg), (ii) musculoskeletal injuries, (iii) neurological, mental, and cardiovascular disorders or diseases, (iv) medication with central nervous or cardiovascular effects, (v) pregnancy, and (vi) open wounds or sensitive scar tissue at the lower limbs were excluded. Suitable participants gave their written informed consent before participating. The study was approved by the Ethics Committee of the Otto von Guericke University at the Medical Faculty and the University Hospital Magdeburg (155/24) in accordance with the principles of the Declaration of Helsinki on human experimentation.

### Experimental procedure

In a randomized and counterbalanced cross-over trial, participants completed one experimental session. Upon arrival, participants’ anthropometric data, resting blood pressure (i.e., systolic and diastolic blood pressure), as well as limb circumference and skinfold thickness of both thighs were determined. Afterwards, the AOP of the lower extremities was determined during six conditions (i.e., both unilateral and bilateral inflation during supine, seated, and standing position) with a rest period of 5 min between measurements to ensure restoration of homeostasis after movement (Jessee et al., 2016; Hughes et al., 2018). The 5-min rest period started after the position for the upcoming measurement had been taken. The order of the conditions as well as the leg in which AOP was determined was randomized with the integer set generator using random.org. The conditions were randomized as follows: randomization of (i) the order of body positions (i.e., supine-seated-standing, supine-standing-seated, seated-supine-standing, seated-standing-supine, standing-supine-seated, or standing-seated-supine), (ii) the extremity in which the AOP was measured during each body position (i.e., left or right), and (iii) the order of the inflation conditions (i.e., unilateral-bilateral or bilateral-unilateral tourniquet cuff inflation). Prior to each condition, two 10 x 76 cm pneumatic tourniquet cuffs (UT 1330-L, ulrich GmbH & Co. KG, Ulm, Germany) were applied to the most proximal part of both legs and connected to an automatic tourniquet system (Heidi™, ulrich GmbH & Co. KG, Ulm). The minimal adjustable pressure of the respective device is ± 5 mmHg with a pressure accuracy of ± 3 mmHg according to the manufacturer’s specifications (Hughes et al., 2025). To assess arterial blood flow, a handheld, bidirectional Doppler probe (Dopplex DMX, Huntleigh Healthcare Ltd, Cardiff, UK) was placed over the posterior tibial artery of the respective leg with an insonation angle between 45-60° against the direction of arterial blood flow according to the manufacturer’s manual. For the exact position, the Doppler probe was placed at the most prominent point of the medial malleolus. The probe was then slowly moved posteriorly until the pulsatory signal was most clearly audible. The respective points were marked with washable felt-tip pen. The tourniquet cuff was gradually inflated until blood flow could no longer be detected. The detection was based on acoustic as well as visual feedback on a small display presenting the blood flow as a curve. The inflation protocol was performed in accordance with several previous studies (Sieljacks et al., 2018; Spitz et al., 2019; Evin et al., 2021). Initially, the tourniquet cuff was inflated to 50 mmHg and stepwise gradually increased by 10 mmHg (with holds) every 10 s until blood flow was no longer detectable (Tafuna'i et al., 2021; Evin et al., 2021; Vehrs et al., 2023) by acoustic and visual feedback. The AOP was defined as the first 10 mmHg step during which the blood flow could no longer be detected. During unilateral tourniquet cuff inflation, only the tourniquet cuff at the leg that was measured was inflated, while during bilateral tourniquet cuff inflation, both tourniquet cuffs were inflated simultaneously. Considering the utilized inflation protocol, a practical meaningful difference in AOP between unilateral and bilateral tourniquet cuff inflation was defined as ≥ 10 mmHg. AOP determination was always performed in the same leg within one condition. All measurements were performed by one assessor. It must be noted that the experimental setup did not allow for blinding the assessor who determined the AOP during unilateral and bilateral tourniquet cuff inflation. In the supine position, the participants were lying on a therapy table with their arms resting at their sides. In the seated position, the participants were sitting upright with 90° hip and knee flexion angles. In standing position, the participants were standing upright with straight legs slightly leaning against the wall. At the beginning (i.e., before the first tourniquet cuff inflation) and end of each condition (i.e., when blood flow was no longer detectable right before tourniquet cuff deflation), systolic and diastolic blood pressure was measured auscultatory in the left arm using a sphygmomanometer while heart rate was assessed via heart rate monitor attached to a chest strap (Vantage V2, Polar Electro GmbH Deutschland, Berlin, Germany). According to Angius et al. (2022), mean arterial pressure was calculated using following formula:


$$\text{Mean}\;\text{arterial}\;\text{pressure}=\;\frac{(2\times \text{diastolic}\;\text{blood}\;\text{pressure})+\text{systolic}\;\text{blood}\;\text{pressure}}{3}$$

Participants were instructed to (i) refrain from consumption of alcohol and analgesics for 24 h and strenuous exercise for 48 h as well as (ii) have the last meal and caffeine intake at least 2 and 8 h, respectively, prior to the laboratory visit.

### Statistical analysis

Data were checked for normal distribution using the Shapiro-Wilk test (**Supplementary Table 1**). However, given that rmANOVAs have been shown to be robust to the violation of the normality (Blanca et al., 2017), non-parametric tests were not used. Therefore, an inflation condition [uni-, bilateral] × body position [supine, seated, standing] rmANOVA was conducted for AOP. For heart rate and mean arterial pressure, an inflation condition [uni-, bilateral] × body position [supine, seated, standing] × time [start, end] rmANOVA was performed. Greenhouse-Geisser correction was applied if sphericity was violated. In case of significant interaction or main effects, *post-hoc* tests with Bonferroni corrections were performed. The effect sizes were determined by calculating partial eta squared (*η_p_^2^*) and Cohen’s *d* (*d*), which were interpreted according to Cohen (2013): 0.01 and 0.2 = small, 0.06 and 0.5 = medium, and 0.14 and 0.8 = large effect, respectively. Mean differences (MD) and 95% confidence intervals (95% CI) are presented and the level of significance was set at *p* ≤ 0.05. All statistical analyses were conducted using JASP Statistics (version 0.19.3, University of Amsterdam, Amsterdam, Netherlands).

## Results

A total number of 25 participants (**Table 1**) completed all measurements. The assessment of two participants had to be repeated since adverse events occurred during the first laboratory visit. Both participants felt uncomfortable and experienced dizziness during bilateral tourniquet cuff inflation in standing position. The cuff was deflated immediately and the participants moved into supine position until their symptoms disappeared. The measurement was repeated after 7 days. Furthermore, one value for heart rate was missing due to technical issues at the beginning of unilateral tourniquet cuff inflation during seated position (1 male). There was a significant difference between limbs for skinfold thickness (*p* = 0.045, *d* = 0.42) but not for thigh circumference (*p* = 0.583).

**Table 1:** Participants’ characteristics expressed as means ± standard deviations as well as range (min-max).

Characteristics		N = 25 (17 males, 8 females)
Age (yrs)		24 ± 4^*^ 15 (21-36)
Weight (kg)		73.4 ± 10.7 39.2 (55.0-94.2)
Height (cm)		176.0 ± 9.1^*^ 29.5 (158.0-187.5)
Body mass index (kg · m^-2^)		23.6 ± 2.3 8.7 (19.0-27.7)
Limb circumference (cm)	Left thigh	55.8 ± 5.5^*^ 26.0 (48.0-74.0)
	Right thigh	55.7 ± 5.5^*^ 26.0 (48.0-74.0)
Skinfold thickness (mm)	Left thigh	15.1 ± 9.0 33.0 (1.0-34.0)
	Right thigh	16.1 ± 9.8^#^ 33.0 (2.0-35.0)
Systolic blood pressure (mmHg)		118.9 ± 10.1 37.0 (102.0-139.0)
Diastolic blood pressure (mmHg)		69.6 ± 6.1 22.0 (60.0-82.0)

^*^ Not normally distributed.

^#^ Significant difference to left thigh (paired sample *t*-test).

### Arterial occlusion pressure

**Figure 1:**
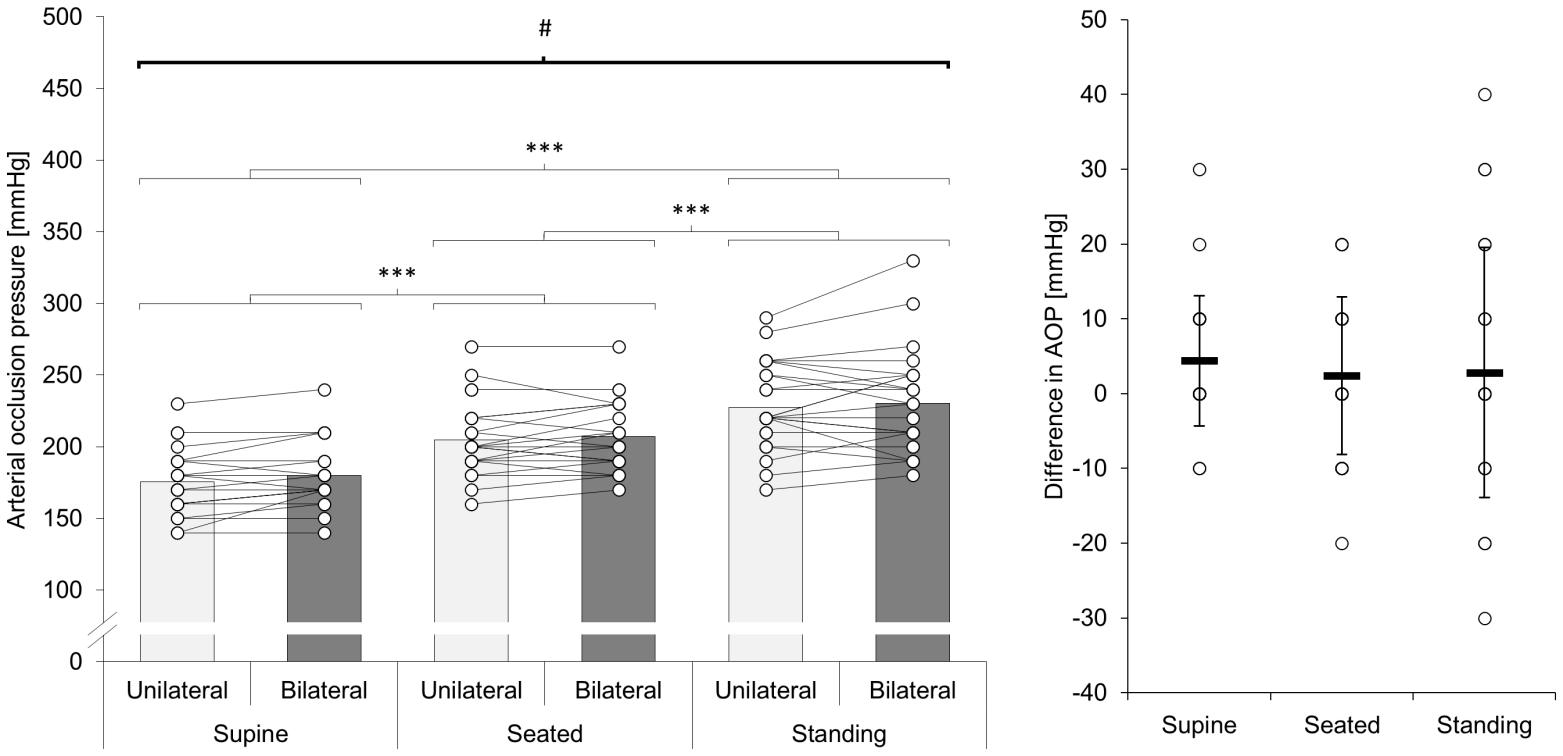
Means and individual data points for arterial occlusion pressure determination with unilateral and bilateral tourniquet cuff inflation in a supine, seated, and standing position. Significant difference of the main inflation condition effect is expressed as ^#^*p* < 0.05. Significant differences between body positions are marked with ^*^p < 0.05, ^**^p < 0.01, and ^***^p < 0.001.

There was no interaction (*F* = 0.147, *p* = 0.820, *η_p_^2^* = 0.006) but a main effect of inflation condition (*F* = 9.071, *p* = 0.006, *η_p_^2^* = 0.274) and body position (*F* = 75.496, *p* < 0.001, *η_p_^2^* = 0.759). *Post-hoc* analysis revealed that AOP was higher during the bilateral compared with the unilateral measurement (*MD* = 3.2 mmHg [95% CI = 1.0-5.4], *p* = 0.006, *d* = 0.12) regardless of body position. Furthermore, regardless of inflation condition, AOP was higher in standing compared to seated (*MD* = 23.0 mmHg [95% CI = 9.8-36.2], *p* < 0.001, *d* = 0.85) and supine position (*MD* = 51.2 mmHg [95% CI = 40.7-61.7], *p* < 0.001, *d* = 1.90). Additionally, AOP was higher in the seated compared to supine position (*MD* = 28.2 mmHg [95% CI = 20.4-36.0], *p* < 0.001, *d* = 1.04). Descriptive data of AOP are shown in **Figure 1** and **Table 2**.

### Heart rate

No interactions (inflation condition × body position × time: *F* = 0.682, *p* = 0.511, *η_p_^2^* = 0.029, inflation condition × body position: *F* = 2.365, *p* = 0.105, *η_p_^2^* = 0.093, inflation condition × time: *F* = 1.068, *p* = 0.312, *η_p_^2^* = 0.044, and body position × time: *F* = 2.412, *p* = 0.101, *η_p_^2^* = 0.095) or main effects of inflation condition (*F* = 0.022, *p* = 0.884, *η_p_^2^* < 0.001) and time (*F* = 0.901, *p* = 0.352, *η_p_^2^* = 0.038) were found for heart rate. However, there was a main effect of body position (*F* = 87.014, *p* < 0.001, *η_p_^2^* = 0.791) and *post-hoc* analysis revealed that heart rate was higher in standing compared to seated (*MD* = 12.8 bpm [95% CI = 8.4-17.2], *p* < 0.001, *d* = 1.14) and supine position (*MD* = 21.6 bpm [95% CI = 16.4-26.9], *p* < 0.001, *d* = 1.92), regardless of inflation condition and time. In addition, heart rate was higher in seated compared to supine position (*MD* = 8.8 bpm [95% CI = 6.1-11.6], *p* < 0.001, *d* = 0.78). Descriptive data of heart rate are presented in **Table 2**.

**Table 2:** Means ± standard deviations of arterial occlusion pressure, heart rate, and blood pressure during determination of arterial occlusion pressure during unilateral and bilateral tourniquet cuff inflation in a supine, seated, and standing position.

Physiological parameters		Supine		Seated		Standing	
		Unilateral	Bilateral	Unilateral	Bilateral	Unilateral	Bilateral
Arterial occlusion pressure [mmHg]		175.6 ± 22.0	180.0 ± 22.0	204.8 ± 25.2	207.2 ± 23.9	227.6 ± 30.6	230.4 ± 35.6
Heart rate [bpm]	Beginning	63.4 ± 8.2	62.6 ± 7.5	72.8 ± 10.2	70.4 ± 9.4	82.5 ± 13.6	83.9 ± 13.8
	End	62.0 ± 9.7	60.5 ± 8.5	70.1 ± 10.2	70.4 ± 10.0	83.1 ± 16.3	85.4 ± 14.4
Mean arterial pressure [mmHg]	Beginning	83.8 ± 5.2	83.5 ± 4.9	86.3 ± 5.8	85.6 ± 4.4	85.5 ± 6.2	84.5 ± 5.7
	End	84.0 ± 5.1	84.6 ± 5.1	83.9 ± 9.4	85.8 ± 5.5	85.1 ± 7.1	86.6 ± 6.8

### Mean arterial pressure

There were no interactions (inflation condition × body position × time: *F* = 0.465, *p* = 0.631, *η_p_^2^* = 0.019, inflation condition × body position: *F* = 0.197, *p* = 0.822, *η_p_^2^* = 0.008, inflation condition × time: *F* = 3.009, *p* = 0.096, *η_p_^2^* = 0.111, and body position × time: *F* = 2.713, *p* = 0.076, *η_p_^2^* = 0.102) or main effects (inflation condition: *F* = 0.985, *p* = 0.331, *η_p_^2^* = 0.039, body position: *F* = 1.238, *p* = 0.299, *η_p_^2^* = 0.049, and time: *F* = 0.047, *p* = 0.831, *η_p_^2^* = 0.002) for mean arterial pressure. Descriptive data of blood pressure are provided in **Table 2**.

## Discussion

To the authors’ knowledge, the present study compared the AOP measured in the lower limbs during unilateral and bilateral tourniquet cuff inflation in different body positions for the first time. The AOP was found to be higher during bilateral compared with unilateral tourniquet cuff inflation regardless of the body position. However, considering the defined practically meaningful difference according to the used inflation protocol (i.e., ≥ 10 mmHg) in combination with the small difference (i.e., 3.2 mmHg) and trivial effect size (i.e., *d* = 0.12), the results indicate that the differences in AOP between unilateral and bilateral measurement might be negligible. This is further supported by the fact that the *MD* between conditions is similar to the pressure accuracy (i.e., ± 3 mmHg) of the used device (according to manufacturer’s manual) with the minimum adjustable pressure of ± 5 mmHg. In this regard, results by Hughes et al. (2021) found similar differences in mean AOP between legs (e.g., right: 199 ± 15 mmHg versus left: 194 ± 22 mmHg) as well as between days within legs (e.g., 199 ± 15 mmHg versus 201 ± 20 mmHg) without being statistically significant (*p* > 0.05). However, the differences in AOP between inflation conditions (i.e., unilateral and bilateral tourniquet cuff inflation) in the present study ranged from 0–40 mmHg with four cases revealing a difference of 30 mmHg (13.6 – 21.4%) and one case with a difference of 40 mmHg (13.8%). This supports the recommendation by Queiros et al. (2023) to perform the measurements anyway since some individuals may exhibit pronounced differences, for example the difference of 80 mmHg in AOP between legs in one participant in the study by Tafuna'i et al. (2021). Therefore, the results must be verified also for cohorts in which differences are more likely to occur (e.g., patients with cardiovascular disease or limb conditions).

The present results are supported by the physiological responses during measurements since no differences in cardiovascular responses have been observed between unilateral and bilateral tourniquet cuff inflation. This is in contrast to our hypothesis, since it was expected that the bilateral venous expansion during bilateral tourniquet cuff inflation would lead to a higher mechano-metaboreflex activation and hemodynamic measures. However, longer occlusion periods might be required for this, given that Angius et al. (2022) occluded blood flow completely for 3 min to trigger metaboreflex activation with significant hemodynamic responses. Furthermore, participants additionally performed a resistance exercise consisting of dynamic contractions (unilateral knee extensions at 70% of *W*_max_, 30 repetitions per min) for 3 min (Angius et al., 2022), suggesting a significant accumulation of metabolites and deoxygenation in the muscle prior to the occlusion protocol. Therefore, the present results might be related to (i) the short duration of the cuff inflation and total occlusion time as well as (ii) the physical inactivity to induce a significant muscle ischemia or venous expansion and therefore depolarize group III/IV afferent fibers (Husmann et al., 2018). Thus, given the trivial effect size, it seems more probable that the small difference was related to random fluctuation. In this regard, the stepwise inflation of 10 mmHg might have biased the results, since even small fluctuations between measurements are expressed as at least a 10-mmHg difference. This suggests that the differences might be related to the used inflation protocol.

It was further found that the AOP was highest in standing followed by the seated and the supine position. This finding corresponds to those of other studies investigating the influence of body position on AOP (Hughes et al., 2018; Sieljacks et al., 2018; Queiros et al., 2024) highlighting the influence of hydrostatic pressure on peripheral blood flow (Wilkins et al., 1950; Hinghofer-Szalkay, 2011) contributing to changes in AOP with increasing upright posture of the body (Queiros et al., 2023). In addition, heart rate increased progressively from supine to seated to standing position. Such changes might have contributed to the increase in AOP across these positions, especially in the lower limbs (Sieljacks et al., 2018).

### Limitations

There are some limitations that need to be considered when interpreting the results of the current study. As a first limitation, the present trial only measured AOP in young healthy male and female sport science students. Therefore, the homogeneity of the current sample limits transfer of findings particularly to clinical populations (e.g., acute and/or chronic intra-individual limb conditions due to injury or surgery). Secondly, as the inflation protocol might influence the resulting AOP (Queiros et al., 2023; Vehrs, 2024), the present results are only valid for the protocol used in the current study. For instance, the stepwise increase of 10 mmHg every 10 s according to the inflation protocol might have limited the resolution to detect very small differences. Smaller steps (e.g., ≤ 5 mmHg) might have led to different results. Third, each AOP measurement was only performed once and not repeated so that intraindividual variations (i.e., coefficient of variation) have not been considered, which might have affected the confidence and interpretation of the present results. However, Hughes et al. (2018) demonstrated excellent reliability for AOP determination during supine (intraclass correlation coefficient (ICC) = 0.982), seated (ICC = 0.975), and standing position (ICC = 0.953) with a coefficient of variation < 3% in the lower extremities using an automatic tourniquet system. Other studies have also found good to excellent test-retest reliability for AOP measurements during supine, seated, and standing position (ICC = 0.90, ICC = 0.873, and ICC = 0.858, respectively) in the upper body using doppler ultrasound (Karanasios et al., 2022). Nevertheless, future studies should perform multiple measurements to calculate coefficients of variation and/or ICCs to strengthen the meaningfulness of their results. As another limitation, heart rate and blood pressure were recorded at the beginning and the end of each AOP measurement, which might have influenced the sensitivity and therefore limited informative value to support underlying mechanisms. Finally, the sample size calculation was conducted with an assumed medium effect size (*f* = 0.25). However, it must be noted that the current results demonstrated only a trivial effect (*d* = 0.12).

## Conclusion

Although there was a statistically significant difference in AOP between unilateral and bilateral tourniquet cuff inflation regardless of body position, it might not be of practical relevance based on its magnitude indicated by the trivial effect size. Therefore, it seems to have only minor impact whether the tourniquet cuff(s) is/are inflated unilaterally or bilaterally during AOP determination when prescribing individual tourniquet cuff pressure for bilateral BFR exercise for the lower extremities in young healthy individuals using this specific BFR device and AOP measurement protocol (Hughes et al., 2025). However, since there have been some substantial differences between AOP during unilateral and bilateral tourniquet cuff inflation (i.e., ≥ 30 mmHg), practitioners should still consider measuring AOP during bilateral tourniquet cuff inflation when performing bilateral exercise for safety reasons. In this regard, practitioners should also be aware of potential adverse events during bilateral cuff inflation in a standing position.

Finally, future trials with different designs (e.g., inflation protocol with smaller gradations), devices (e.g., automatic AOP determination), and populations (e.g., patients with cardiovascular disease or limb conditions) are needed to further investigate the practical and clinical relevance of bilateral tourniquet cuff inflation during AOP determination. Furthermore, future research should also consider conducting an equivalence trial rather than a comparison study on unilateral vs bilateral cuff inflation during AOP determination since differences were small with a trivial effect.

## Data Availability

The raw data supporting the conclusions of this article will be made available by the authors, without undue reservation.
