# Supplementary material for: Does exercising bilaterally require measuring bilaterally? Bilateral versus unilateral tourniquet cuff inflation during measurement of arterial occlusion pressure: considerations for blood flow restriction exercise
Source: Front Physiol. 2026 Jun 5;17:1789582. doi: 10.3389/fphys.2026.1789582 (PMC13278906; doi:10.3389/fphys.2026.1789582)
Supplement: Supplementary file 1 [file Table1.docx]

**Table S1.** Level of significance (*p*) resulting from the Shapiro-Wilk test for arterial occlusion pressure, heart rate, and mean arterial pressure during determination of arterial occlusion pressure with unilateral and bilateral tourniquet cuff inflation in a supine, seated, and standing position. Significant values indicating a non-normal distribution of data are displayed in **bold**.

|  | | **Supine** | | **Seated** | | **Standing** | |
| --- | --- | --- | --- | --- | --- | --- | --- |
|  |  | **Unilateral** | **Bilateral** | **Unilateral** | **Bilateral** | **Unilateral** | **Bilateral** |
| **Arterial occlusion pressure [mmHg]** | | .429 | **.021** | .117 | .121 | .529 | .060 |
| **Heart rate [bpm]** | **Beginning** | .388 | .638 | .186 | .147 | **.007** | .292 |
|  | **End** | .686 | .828 | **.043** | .612 | .118 | .288 |
| **Mean arterial pressure [mmHg]** | **Beginning** | .701 | .380 | **.028** | .446 | .510 | .056 |
|  | **End** | .619 | .625 | **< .001** | .140 | .790 | .496 |
